# IL-33 Is Essential for Adjuvant Effect of Hydroxypropyl-β-Cyclodexrin on the Protective Intranasal Influenza Vaccination

**DOI:** 10.3389/fimmu.2020.00360

**Published:** 2020-03-06

**Authors:** Shingo Kobari, Takato Kusakabe, Masatoshi Momota, Takayuki Shibahara, Tomoya Hayashi, Koji Ozasa, Hideaki Morita, Kenji Matsumoto, Hirohisa Saito, Shuichi Ito, Etsushi Kuroda, Ken J. Ishii

**Affiliations:** ^1^Laboratory of Adjuvant Innovation, Center for Vaccine and Adjuvant Research, National Institutes of Biomedical Innovation, Health and Nutrition (NIBIOHN), Osaka, Japan; ^2^Department of Pediatrics, Graduate School of Medicine, Yokohama City University, Kanagawa, Japan; ^3^Laboratory of Mock-up Vaccine Project, Center for Vaccine and Adjuvant Research, National Institutes of Biomedical Innovation, Health and Nutrition (NIBIOHN), Osaka, Japan; ^4^Laboratory of Vaccine Science, WPI Immunology Frontier Research Center (IFReC), Osaka University, Osaka, Japan; ^5^Division of Vaccine Science, Department of Microbiology and Immunology, The Institute of Medical Science, The University of Tokyo, Tokyo, Japan; ^6^International Research and Development Center for Mucosal Vaccines, The Institute of Medical Science, The University of Tokyo, Tokyo, Japan; ^7^Department of Allergy and Clinical Immunology, National Research Institute for Child Health and Development, Tokyo, Japan; ^8^Department of Immunology, Hyogo College of Medicine, Hyogo, Japan

**Keywords:** IL-1α, IL-13, lung, DAMPs, ST2, AT2

## Abstract

Vaccine adjuvants are traditionally used to augment and modulate the immunogenicity of vaccines, although in many cases it is unclear which specific molecules contribute to their stimulatory activity. We previously reported that both subcutaneous and intranasal administration of hydroxypropyl-β-cyclodextrin (HP-β-CD), a pharmaceutical excipient widely used to improve solubility, can act as an effective adjuvant for an influenza vaccine. However, the mechanisms by which mucosal immune pathway is critical for the intranasal adjuvant activity of HP-β-CD have not been fully delineated. Here, we show that intranasally administered HP-β-CD elicits a temporary release of IL-33 from alveolar epithelial type 2 cells in the lung; notably, IL-33 expression in these cells is not stimulated following the use of other vaccine adjuvants. The experiments using gene deficient mice suggested that IL-33/ST2 signaling is solely responsible for the adjuvant effect of HP-β-CD when it is administered intranasally. In contrast, the subcutaneous injection of HP-β-CD and the intranasal administration of alum, as a damage-associated molecular patterns (DAMPs)-inducing adjuvant, or cholera toxin, as a mucosal adjuvant, enhanced humoral immunity in an IL-33-independent manner, suggesting that the IL-33/ST2 pathway is unique to the adjuvanticity of intranasally administered HP-β-CD. Furthermore, the release of IL-33 was involved in the protective immunity against influenza virus infection which is induced by the intranasal administration of HP-β-CD-adjuvanted influenza split vaccine. In conclusion, our results suggest that an understanding of administration route- and tissue-specific immune responses is crucial for the design of unique vaccine adjuvants.

## Introduction

Vaccines are one of the most effective preventive measures for diseases. However, as the threat of infectious diseases diminishes, vaccine safety is increasing in importance, particularly in developed countries. Subunit vaccines that use more purified antigens have a high safety profile but are often poorly immunogenic. To improve the immune response to subunit vaccines, vaccine adjuvants have been actively developed in recent years (1–3). Unfortunately, many of the candidate adjuvants proved too reactogenic and were poorly tolerated, so they did not advance further into clinical development. At present, there are just a few US Food and Drug Administration (US-FDA)-approved adjuvants. Furthermore, despite widespread research on vaccine adjuvants, there is little known regarding the pathways affected by many potential adjuvants. Pattern-associated molecular patterns (PAMPs), acting via pathogen-recognition receptors (PRRs), such as Toll-like receptors (TLRs), cytosolic nucleotide oligomerization domain-like receptors (NODs), retinoic acid-inducible gene-based-I (RIG-I)-like receptors (RLRs), and C-type lectin-like receptors (CLRs), are one of category of immunopotentiating molecules, and they have been widely employed in preclinical and clinical studies (4). In contrast, the precise roles of innate recognition for other adjuvants, such as alum (despite being the most widely adjuvant), emulsions, and particulates are less characterized. To rationally design vaccines against various diseases and obtain information on adjuvant safety, we need to understand the mechanisms involved in adjuvant-enhanced immunity.

In recent years, mucosal immunity has attracted a lot of attention, and there has been a corresponding focus on the mucosal route of vaccination (5–7). However, attempts to develop mucosal vaccines have not yet been successful, in part because they require adjuvants suitable for characteristic mucosal environments to obtain adequate vaccine effects (8, 9). Recently, we reported that hydroxypropyl-β-cyclodextrin (HP-β-CD) has potent adjuvant activities in mice and cynomolgus monkeys (10–12). In those studies, we found that the intranasal administration of HP-β-CD can enhance the secretion of antigen-specific IgG and IgA into the serum and the airway fluid and the administration of an intranasal flu vaccine adjuvanted with HP-β-CD protected mice against a lethal challenge with influenza virus. Additionally, it was suggested that enhanced humoral immunity by intranasally administered HP-β-CD was mediated by MyD88, which is the downstream signaling molecule for TLRs, but also for IL-1 family cytokines including IL-1 and IL-33. Although HP-β-CD is thought to show the adjuvanticity by stimulating the release of damage-associated molecular patterns (DAMPs) because temporary release of double-strand DNA was detected after intranasal and subcutaneous injection, the detailed mechanism of adjuvanticity for intranasally administered HP-β-CD remains poorly understood.

Here, we found that IL-33, but not IL-1 is released from alveolar epithelial cells following the intranasal administration of HP-β-CD and that IL-33-mediated signaling was essential for its adjuvanticity. Moreover, we found completely different mechanisms of action between intranasally administered and subcutaneously administered HP-β-CD.

## Materials and Methods

### Antigens and Adjuvants

Ovalbumin protein (OVA; Kantokagaku, Tokyo, Japan) was used as a model antigen. HP-β-CD (ISP Technologies, Assonet, MA, USA), Alhydrogel (alum; Invivogen, San Diego, CA, USA), cholera toxin (CT), cholera toxin B subunit (CTB) (Wako, Osaka, Japan) and recombinant mouse IL-33 (R&D systems, Minneapolis, MN, USA) were used as adjuvants. Influenza split vaccine (SV) was manufactured with egg-based technology. SV contains influenza virus hemagglutinin (HA) surface Ag from New Caledonia/20/1999 (H1N1) (The Research Foundation for Microbial Diseases of Osaka University) as previously described (11).

### Mice

C57BL/6J mice were obtained from CLEA Japan, Inc. (Tokyo, Japan). *Tlr4* ^−/−^, *Tlr7*^−/−^, *Tlr9* ^−/−^, *Il33*^−/−^, *Il1r1*^−/−^, *St2*^−/−^, and *Il13*^−/−^ mice were kindly provided by Dr. Akira (13–15), Dr. Nakanishi (16), Dr. Ziegler (17), and Dr. McKenzie (18). *Tnf/Tbk1*-knockout mice were prepared as described (19). *Il1r1*^−/−^ mice were purchased from the Jackson Laboratory (Maine, U.S.A.). These deficient mice were bred on a C57BL/6 background and housed under specific pathogen-free conditions in the animal facility of the National Institute of Biomedical Innovation, Health and Nutrition (NIBIOHN). Mice were age- and sex-matched and were 6–9 weeks old when used in experiments. The experiments with *Il13*^−/−^ mice were performed in National Research Institute for Child Health and Development (NCCHD). All animal experiments were performed according to the guidelines for the care and use of laboratory animals established by NIBIOHN and NCCHD.

### Immunization of Mice and Preparation of Lung Washes and Serum

Antigens and adjuvants were each dissolved in PBS. In the vaccination model, mice were immunized intranasally or subcutaneously twice at 2 week intervals, with a solution containing 10 μg of OVA with or without adjuvant. Nasal and subcutaneous administration volumes were 15 μl into each nostril and 200 μl into the dorsal flank, respectively. All immunizations were performed under anesthetic (ketamine and xylazine). Blood and bronchoalveolar lavage fluid (BALF) were taken on day 28. Blood was collected using heparinized capillary tubes and centrifuged at 2,500 × *g* for 5 min. The plasma was then collected and stored frozen at −40°C until use. BALF samples were obtained by washing the lung with 0.7 + 0.5 ml of PBS. Lung wash samples were centrifuged at 9,000 × *g* for 10 min. The resulting supernatants were collected and stored frozen at −40°C until the measurement of antibodies and cytokines. HP-β-CD or recombinant IL-33 were administered at 10% w/w or 100 ng, respectively. Alum was administered at 100 μg per mouse. CT was used after adding 1 μg of CTB to 1 ng of CT per mouse.

### Measurement of Cytokines in BALFs and Lung Homogenate Supernatants

To evaluate the cytokines IL-33 and IL-1α in BALF, mice were injected intranasally with PBS with or without HP-β-CD, alum, or CT. For the collection of BALF, mice were euthanatized, and their lungs were lavaged twice with consecutive 500 μl instillations of PBS. BALFs were collected at 0, 2, 6, 12, and 24 h after adjuvant administration. The collected BALFs were centrifuged at 2,500 × *g* for 5 min at 4°C, and the resulting supernatants were stored at −40°C for later use in the measurement of cytokine levels. For the collection of lung lysates, mice were euthanatized, and their lungs were excised and homogenized in 10 ml of PBS using a gentleMACS™ Dissociator (Miltenyi Biotec, Bergisch Gladbach, NRW, Germany). The lung homogenates were centrifuged at 300 × *g* for 5 min at 4°C, and the resulting supernatants were then centrifuged at 9,000 × *g* for 5 min at 4°C. The new supernatants were stored at −40°C for later use in the measurement of cytokine levels. The levels of IL-1α were measured using an ELISA kit (BioLegend, San Diego, CA, USA) in accordance with the manufacturer's instructions. The levels of IL-33 were measured using the following method. Briefly, 96-well plates were coated with 2 μg/ml purified anti-mouse IL-33 antibodies (clone Poly5165; BioLegend) in PBS overnight at 4°C. They were then washed with PBS containing 0.05% Tween-20 (PBST) and incubated for 1 h with blocking buffer (RPMI containing 5% FCS). After blocking, the plates were washed and incubated with diluted BALF or recombinant IL-33 as a standard overnight at 4°C. They were then washed again and incubated for 1 h with 0.5 μg/ml biotin-conjugated anti-mouse IL-33 antibody (clone Poly5165; BioLegend) in PBS with 1% BSA. The plates were then washed again and incubated for 20 min with horseradish peroxidase (HRP)-conjugated avidin. After a final wash, the samples were incubated with a reagent from the TMB Microwell Peroxidase Substrate System (KPL, Gaitherburg, MD, USA) to initiate the color reaction, in accordance with the manufacturer's protocol. The reaction was stopped by the addition of 2 N H_2_SO_4_, and the optical density was measured at a wavelength of 450 nm (OD_450_). Protein concentrations in the supernatant were quantified with the Pierce BCA Protein Assay Kit (Thermo Fisher, Waltham, MA, USA). Total RNA from mouse lung homogenates was extracted with TRIzol LS Reagent (Thermo Fisher, Waltham, MA, USA) and RNeasy Mini Kit (QIAGEN, Venlo, Netherlands). RNA was reverse-transcribed with ReverTra Ace (TOYOBO, Osaka, Japan). The expression of genes was quantified with LightCycler® TaqMan® Master and LightCycler® 480 System (Roche, Penzberg, Germany), according to the manufacturer's instructions. The results are shown as the relative expression standardized to the expression of a gene encoding eukaryotic 18S rRNA. The specific primers and probes used for quantitative RT-PCR were TaqMan probes for Il33 (Cat# 4351372) and 18S rRNA (Applied Biosystems, Waltham, MA, USA).

### OVA-Specific Antibody Response

To measure the OVA-specific total IgG, IgG1, IgG2c, and IgA in the serum and the BALF samples, flat-bottomed 96-well microtiter plates were coated with 10 μg/ml OVA in carbonate buffer overnight at 4°C. The plates were then washed with PBST and incubated for 1 h with blocking buffer (PBST containing 1% BSA). After blocking, the plates were washed and incubated with diluted serum or BALF for 2 h at room temperature. To detect the bound antibody, the plates were washed and incubated for 1 h with HRP-conjugated anti-mouse total IgG, IgG1, IgG2c, or IgA antibody (Southern Biotech, Birmingham, AL, USA). After the plates were washed, enzymatic detection was performed with TMB soluble reagent and terminated by the addition of 2 N H_2_SO_4_. Titers of antigen-specific antibodies were determined by log-linear interpolation of the serum dilution value corresponding to cut-off absorbance (OD450 of 0.2).

### Histological Analysis

To stain the lung tissues, the lungs were inflated with Tissue-Tek optimal cutting temperature (OCT) compound (Sakura, Tokyo, Japan) diluted 1:4 in PBS, subsequently embedded in OCT compound, and frozen at −80°C. Frozen sections (12 μm) of the isolated lung specimens were prepared, fixed, and permeabilized with the BD Cytofix/Cytoperm Plus Fixation/Permeabilization Kit (BD Biosciences) in accordance with the manufacturer's instructions. The lung specimens were incubated overnight at 4°C with purified anti-mouse IL-33 antibodies (clone Poly5165; BioLegend) and anti-pro surfactant protein C (proSP-C) antibodies (EMD Millipore; Merck KGaA, Darmstadt, Germany). After that, we stained them with Alexa Fluor®568 anti-goat IgG and Alexa Fluor®647 anti-rabbit IgG (Invitrogen, San Diego, CA, USA) for 1 h and co-stained them with DAPI (Sigma Aldrich, St. Louis, MO, USA) for 20 min. Images were analyzed with a FluoView (FV10i) confocal microscope (Olympus, Tokyo, Japan).

### Influenza Virus Challenge

The influenza SV (1 μg) or the influenza SV plus HP-β-CD was administered to *Il33*^+/−^ or *Il33*^−/−^ mice at days 0 and 14. Two weeks after the last immunization, the mice were challenged intranasally with 10 × LD_50_ of influenza virus A/PR/8/34. The changes in body weight and the mortality of the challenged mice were monitored for 20 days.

### Statistical Analysis

Statistical analysis was performed using GraphPad Prism6 software (GraphPad Software Inc., LaJolla, CA, USA). The data are shown as the mean ± standard error of the mean (SEM). Means were compared using a *t*-test or one-way ANOVA for multiple groups, and *p* < 0.05 was considered significant.

## Results

### Nasal Administration of HP-β-CD Raises Levels of IL-33, but Not of IL-1α, in BALF

**Figure 1:**
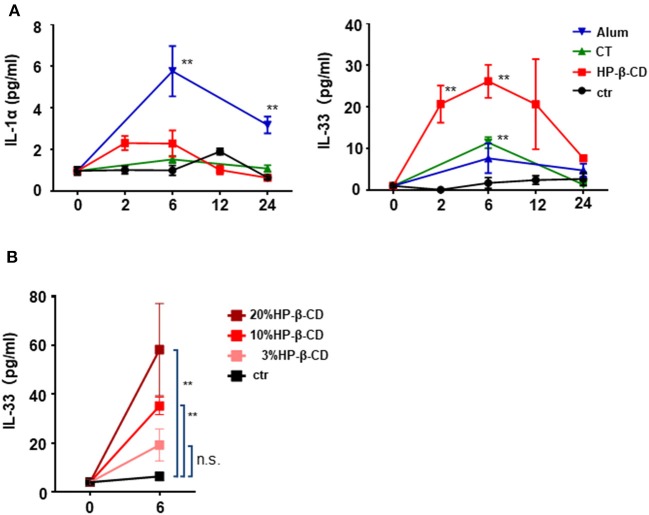
IL-33 in BALF were temporary increased after intranasal administration of HP-β-CD. **(A,B)** IL-1α and IL-33 levels in the BALF of mice intranasally administered a single dose of alum (100 μg/dose), cholera toxin (CT), 10% HP-β-CD, or PBS **(A)** or 3–20% HP-β-CD **(B)**. Cytokine levels in the supernatants of the BALF were measured by ELISA. Results are shown as the mean ± SEM (*n* = 5–6 in each group) and are representative of two experiments. **p < 0.01 compared with mice exposed to PBS (Mann-Whitney U-test).

We previously demonstrated that the adjuvant effect of intranasally administered HP-β-CD disappeared in *Myd88*^−/−^ mice (11). MyD88 is a downstream signaling molecule of the IL-1 family and TLR receptors (20). As the prominent cytokines in the lung, IL-1α is reported to be released from murine alveolar macrophages upon the intratracheal administration of fine particles (21). Moreover, it is well-known that IL-33 is induced during various immune responses in the lung (16, 22). Hence, to investigate whether any cytokines are involved in the adjuvant effect of HP-β-CD, we first performed a time-course analysis of the levels of IL-1α and IL-33 in the BALF after intranasal injection of HP-β-CD. We used alum and cholera toxin (CT) as controls commonly used as a nasal vaccine adjuvant in animal models. Consistent with previous results (21), the BALF levels of IL-1α were increased in mice injected with alum (Figure 1A). In contrast, the BALF IL-33 levels, but not the IL-1α levels, were significantly increased in HP-β-CD-injected mice, particularly around 6 h post-injection (Figure 1A). The observed increase in IL-33 levels completely faded by 24 h. CT administration did not increase the levels of either IL-1α or IL-33 in the BALF. Next, we administered three different concentrations of HP-β-CD to mice. As the concentrations of administered HP-β-CD increased, the level of IL-33 in the BALF also went up (Figure 1B). Collectively, these results clearly indicate that the nasal immunization of HP-β-CD induced an increase in the level of IL-33 protein secreted in the BALF.

### HP-β-CD Induces Robust IL-33 Production in Alveolar Epithelial Type 2 (AT2) Cells

**Figure 2:**
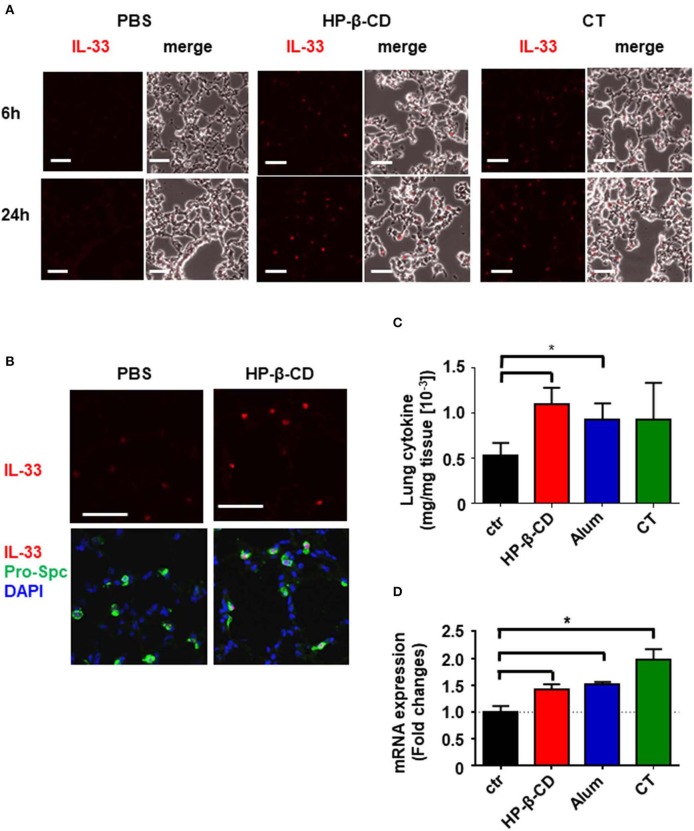
HP-β-CD induced expression of IL-33 from AT2 in the lung. Mice were intranasally administered a single dose of PBS, HP-β-CD, or CT. **(A,B)** Representative images of lung sections collected from these mice at 6 **(A)** or 24 h **(A,B)** after intranasal administration and subjected to immunohistochemistry for IL-33 (red) **(A,B)** and pro-SPC (green) and co-stained with DAPI (blue) **(B)**. White scale bar = 50 μm. **(C,D)** IL-33 protein levels in the supernatants of lung homogenates from 24 h **(C)** and mRNA levels in lung tissues from 6 h **(D)** after intranasal administration with alum, cholera toxin, HP-β-CD, or PBS as determined by ELISA and q-PCR, respectively. Results are shown as the mean ± SEM (*n* = 5–6 in each group) and are representative of two experiments. *p < 0.01 compared with mice exposed to PBS (Mann-Whitney U-test).

It is of note that intranasal administration of mice with HP-β-CD, but not with alum or CT, induced IL-33 secretion in the BALF in 6 h, without inducing IL-1α, while all of the adjuvants were shown to release same amount of dsDNA into BALF 6 h after administration (11). As IL-1α and IL-33 are nuclear cytokines induced in different cell types (16, 21), we thus hypothesized that the cell type(s) that respond to each adjuvant may be different. In order to determine which cells in the lung produced the IL-33 observed in the BALF after an intranasal injection of HP-β-CD, we performed immunohistochemistry to stain IL-33 in the lung tissue at the different time points. Higher level of IL-33 were detected in the lung tissues at 24 h after HP-β-CD administration than at 6 h (Figure 2A) although the peak of IL-33 level in BALF was at 6 h (Figure 1A). Interestingly, CT also increased the level of IL-33 at 24 h (Figure 2A). When we examined the co-localization of IL-33 and alveolar epithelial type 2 (AT2) cells, known to be the major cellular source of IL-33 in murine lungs (16, 18), IL-33 induced by HP-β-CD was restricted to pro-surfactant protein C (pro-SPC)-positive AT2 cells (Figure 2B), suggesting that IL-33 released by HP-β-CD may be released from the AT2 cells.

We previously reported that TANK-binding kinase 1 (TBK1), a cytosolic kinase that is essential for the activation of nucleic acid-dependent downstream signaling, is involved in the induction of immune responses resulting from intranasal HP-β-CD immunization (10). Moreover, it is reported that IL-33 induction is dependent on TBK1-dependent signaling pathway (22). However, a similar level of IL-33 expression was also observed in lung tissues from intranasally HP-β-CD-administered TBK1-deficient mice (Figure S1). This finding suggests that TBK1 signaling is not involved in IL-33 induction after intranasal HP-β-CD administration. Taken together, these results demonstrate that HP-β-CD induced IL-33 production from AT2 cells in TBK-independent mechanism.

The different pattern of IL-33 level in BALF samples and lung sections attempted us to identify more precise mechanism of HP-β-CD to induce the production of IL-33. Recent results have shown that IL-33 functions as an alarmin following the release into the extracellular space by cellular damage or mechanical injury (23). Thus, IL-33 detected in the BALF after the intranasal administration of HP-β-CD is assumed to reflect only the active form of which was released from the cells. To analyze whether HP-β-CD enhances the production of IL-33 as well as the release of it, we measured the protein level of IL-33 in the supernatant of homogenized lung tissues from the mice intranasally injected with HP-β-CD. In consistent with immunohistochemistry data, the level of IL-33 in the homogenized lung tissue were increased by the intranasal administration of HP-β-CD (Figure 2C). Furthermore, a rapid increase of IL-33 mRNA was also observed after the intranasal administration of HP-β-CD. It is worth to mention that mRNA and protein levels of IL-33 in homogenized lung tissues were also elevated following the intranasal administration of alum or CT while they did not increase IL-33 in BALF (Figures 1A, 2C,D). Taken together, these results suggested that HP-β-CD induces not only the temporal release of IL-33, but also the production in mainly AT2 cells.

### The Adjuvant Effect of HP-β-CD Nasal Administration Disappears in *Il33*^−/−^ Mice

**Figure 3:**
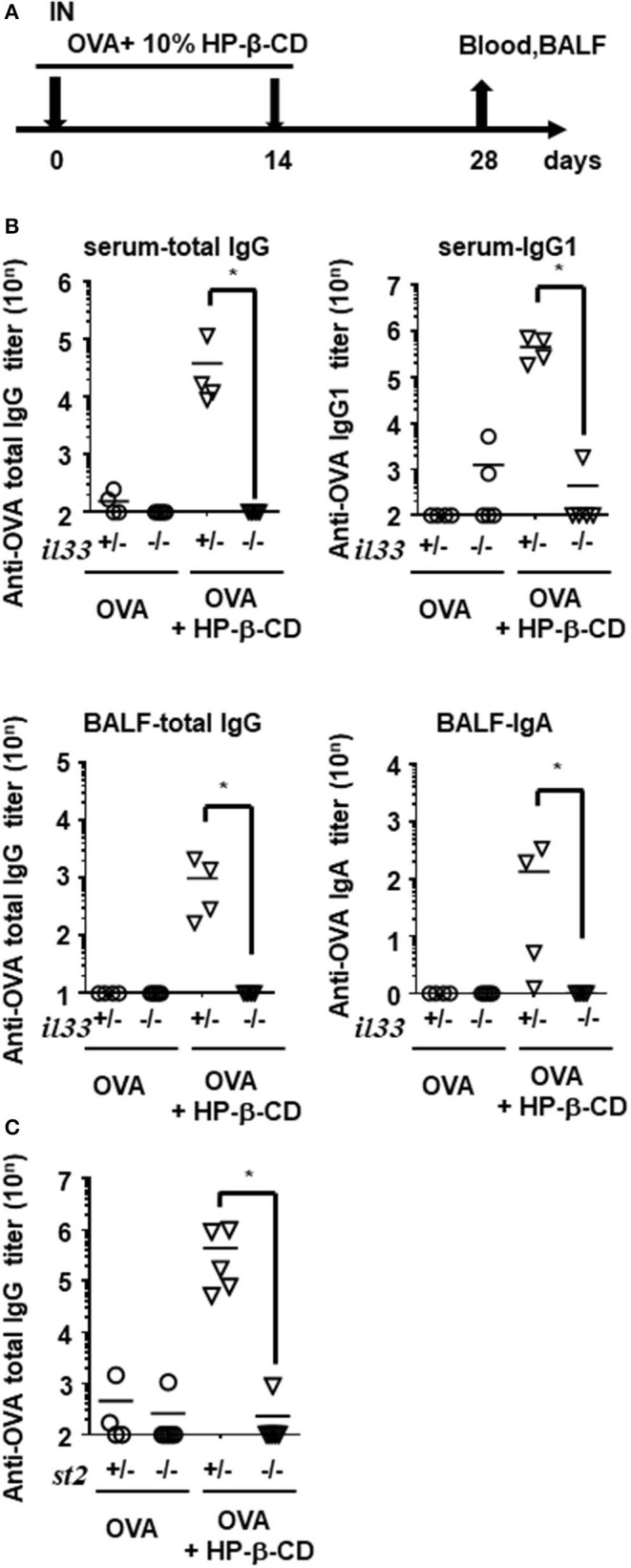
Antibody responses in serum and BALF were hardly increased in HP-β-CD-adjuvanted IL-33 and ST2 knockout mice. **(A)** Time schedules for the vaccination and sampling of blood and BALF. Each dose contained 10 μg of OVA plus 10% HP-β-CD. **(B,C)** *Il33*^+/−^, *Il33*^−/−^ **(B)**, *St2*^+/−^, or *St2*^−/−^ **(C)** mice were immunized with OVA ± HP-β-CD according to the schedule illustrated in **(A)**. Serum and BALF were collected 14 days after the last immunization, and OVA-specific total IgG, IgG1, IgG2c, and IgA levels were measured via ELISAs. The graphs show titers (mean ± SEM, *n* = 5). Data are representative of three independent experiments. *p < 0.01 (Mann-Whitney U-test).

Next, we investigated whether IL-33 plays an important role in HP-β-CD adjuvanticity using innate immune deficient mice. Interestingly, the adjuvanticity of HP-β-CD was distinct from PAMP-driven immune-enhancing effects; OVA-specific IgG titers in *Tlr4*^−/−^, *Tlr7*^−/−^, and *Tlr9*^−/−^ mice after HP-β-CD-OVA vaccination was equivalent to that of wild type (WT) mice (Figure S2A). Therefore, to verify whether IL-33 contributed to the adjuvanticity of HP-β-CD, WT and *Il33*^−/−^ mice were immunized intranasally twice with OVA plus HP-β-CD, and the resulting OVA-specific antibody titers in the serum and BALF were quantified on day 28 post-immunization (Figure 3A). In sharp contrast to WT mice, the adjuvant effect of HP-β-CD completely disappeared in the *Il33*^−/−^ mice, and there was no difference in the OVA-specific antibody titers following the administration of OVA plus HP-β-CD as compared with the administration of OVA alone in these mice (Figure 3B). Additionally, the adjuvanticity was also observed in mice that were immunized with OVA plus recombinant IL-33 instead of HP-β-CD by intranasal administration (Figure S3). These results are consistent with the observation that IL-1 family cytokines, including IL-33, leads to display a mucosal adjuvant effect following their nasal administration (24).

Airway epithelial cells also produce innate cytokines, such as IL-1α, IL-25, and thymic stromal lymphopoietin (TSLP) as well as promote Th2-type immunity. Using a similar experimental set-up, we evaluated the involvement of these cytokines in HP-β-CD adjuvanticity. When HP-β-CD was administered to IL-1α- or TSLP-deficient mice, these mice, like WT mice, exhibited a robust production of anti-OVA total IgG antibody, although the *Il1*α^−/−^ mice had slightly lower levels of it (Figure S2B). Moreover, mice lacking IL-1R1, which is the specific receptor for IL-1α and IL-1β, had levels of anti-OVA total IgG antibody that were comparable with those of WT animals (Figure S2B), whereas mice lacking ST2 (IL-1RL1), the receptor for IL-33, were unable to produce detectable levels of anti-OVA total IgG antibody (Figure 3C). These findings suggest that IL-33/ST2 signaling is more dominant in the adjuvant effect of nasally administered HP-β-CD than other innate cytokine-mediated signaling.

ST2 is expressed on Th2 cells, innate lymphoid type 2 (ILC2) cells, eosinophils, basophils, and mast cells, among others, and they induce the immune responses by producing Th2 cytokines. To investigate the involvement of the major Th2 cytokine IL-13 in the adjuvant effect of HP-β-CD, we nasally administered HP-β-CD to *Il13*^−/−^ mice. The serum anti-OVA antibody titers of HP-β-CD-adjuvanted IL-13-deficient mice were not significantly different from those of similarly treated WT mice, but the anti-OVA IgA antibody titer in the BALF of these mice was significantly lower (Figure S2C). Based on these data together with the data showing that IL-33 has an important role in our model, we concluded that the immune responses that occur after HP-β-CD immunization might be induced by signals from IL-33/ST2 signaling through MyD88.

### Intranasal Administration of HP-β-CD Has a Unique Mode of Action of Adjuvanticity

**Figure 4:**
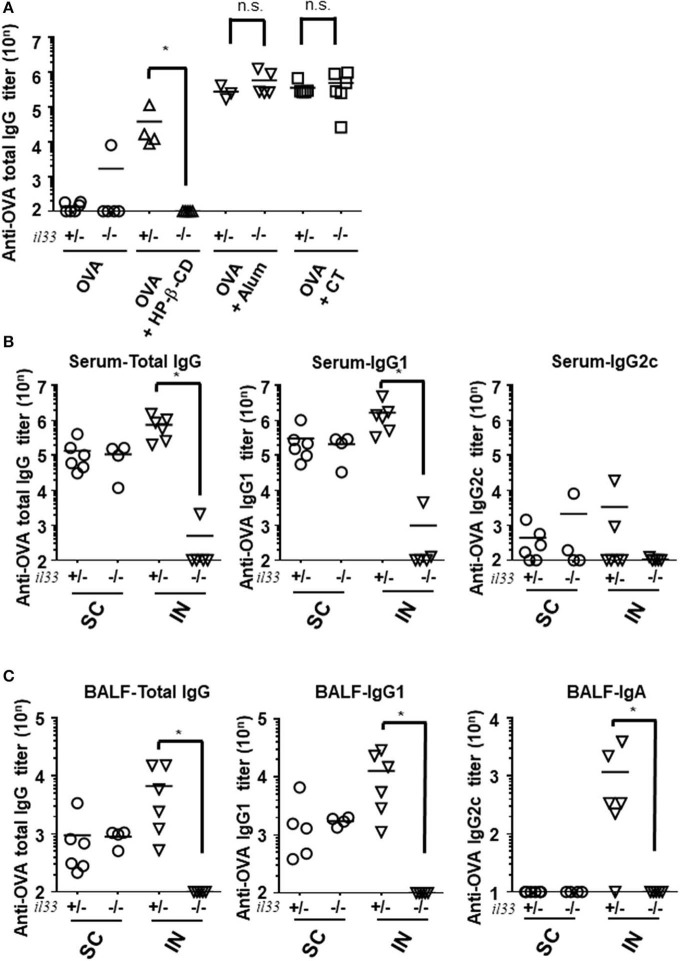
IL-33 signaling affected the adjuvanticity only in intranasal administration of HP-βCD. **(A–C)** *Il33*^+/−^ or *Il33*^−/−^ mice were immunized intranasally **(A–C)** or subcutaneously **(B,C)** with 10 μg of OVA plus 10% HP-β-CD, CT, or 100 μg of alum on days 0 and 14. The anti-OVA total IgG, IgG1, and IgG2c antibody titers in serum **(A,B)** and anti-OVA total IgG, IgG1, and IgA antibody titers in BALF **(C)** were measured via ELISA 14 days after the last immunization. SC, subcutaneous; IN, intranasal. Results are representative of three independent experiments and are shown as the mean ± SEM of 5–6 mice in all groups. *p < 0.05 (Mann-Whitney U-test).

To further characterize the role of IL-33 in adjuvant effects in general, we investigated the requirement for this cytokine following the use of other adjuvants and administration routes. First, we evaluated the antibody responses in alum- or CT- adjuvanted IL-33-deficient mice. Unlike in HP-β-CD-treated *Il33*^−/−^ mice, the antibody responses in the serum and BALF were not decreased in alum- or CT-adjuvanted *Il33*^−/−^ mice compared with similarly treated WT mice (Figure 4A). Second, to explore the possibility that IL-33 might also be involved in the effect of subcutaneous injection with HP-β-CD, we compared the involvement of IL-33 in the antibody responses induced by subcutaneous and intranasal HP-β-CD administration. The induced OVA-specific antibody responses in the serum from WT mice were equivalent between subcutaneously and intranasally HP-β-CD-injected mice. However, in the case of *IL-33*^−/−^ mice injected with HP-β-CD, the production of OVA-specific antibody responses was abolished in the intranasally HP-β-CD-administered mice, whereas the subcutaneously HP-β-CD-immunized mice had robust antibody responses (Figure 4B). In addition, the disappearance of OVA-specific antibody production in these mice occurred not only in the serum (IgG) but also in the BALF (IgA) (Figure 4C). Thus, IL-33 is not required for the adjuvanticity of subcutaneously administered HP-β-CD. These findings were consistent with previous reports that alum induces a completely different response depending on its administration route (21, 25–27). Taken together, these results indicate that stimulation of the IL-33-dependent pathway is unique to the adjuvanticity of intranasally administered HP-β-CD.

### HP-β-CD-Induced IL-33 Enhances Protective Immunity Against PR8 Influenza Virus

**Figure 5:**
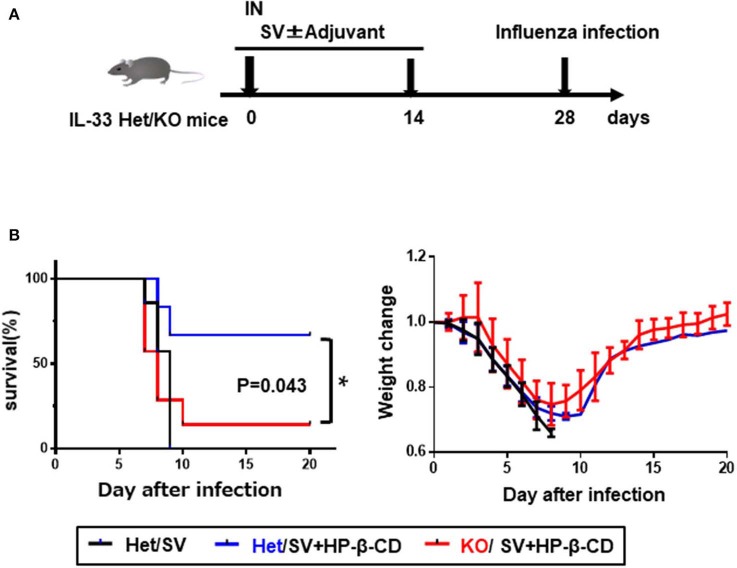
The induction of IL-33 by intranasally HP-β-CD protected against influenza infection. **(A)** Experimental timeline. **(B)** *Il33*^+/−^ or *Il33*^−/−^ mice were vaccinated with the influenza split vaccine (1 μg) in combination with or without HP-β-CD on days 0 and 14. Two weeks after the last immunization, the mice were challenged with 10 × LD_50_ of influenza virus A/PR/8/34. Changes in mortality (left) and body weight (right) were monitored for 20 days. *p < 0.05 [Log-rank (Mantel-Cox) test].

Intranasal vaccination with HP-β-CD as an adjuvant has been previously shown to enhance protection against lethal influenza infection (11). To confirm the protective role of IL-33 during influenza infection, we immunized *Il33*^+/−^ or *Il33* ^−/−^ mice with the influenza split vaccine plus HP-β-CD following PR8 infection (Figures 5A,B). In agreement with the previous report, *Il33*^+/−^ mice were protected against PR8 virus infection following vaccination that was adjuvanted with HP-β-CD. In contrast, the protection provided by this vaccine and adjuvant combination was lost in *Il33*^−/−^ mice. Additionally, compared with WT mice, unvaccinated *Il33*^−/−^ mice showed the same susceptibility to infection. Furthermore, we investigated that HP-β-CD was also able to increase antibody titers against influenza vaccine as well as OVA. Of note, the immune response induced by HP-β-CD was abolished in IL-33 KO mice (Figure S4). These data support the novel finding that the IL-33 induced by the intranasal administration of HP-β-CD can contribute to protection against influenza virus infection.

## Discussion

The purpose of a vaccine is to allow immunocompetent cells to recognize the vaccine antigen(s) when the host is exposed to the corresponding pathogen, so they can induce an appropriate immune reaction. Vaccine development has shifted focus from the classical live-attenuated and inactivated vaccines to subunit vaccines, which consist of purified proteins, emphasize safety, and may also have applications in non-infectious diseases, such as cancer. However, many antigens do not elicit strong immune responses on their own. Consequently, current vaccines often contain adjuvants that can activate the innate immune system to promote the recognition and presentation of antigens by antigen-presenting cells (APCs) and subsequent cell-mediated and humoral immune responses (1). Ligands that themselves activate innate immunity have been studied as vaccine adjuvants, as have damage-associated molecular patterns (DAMPs)-inducing molecules, which cause cell damage and DAMPs release, thus resulting in an enhanced innate immune response (4). However, the mechanisms of action of these adjuvants are poorly understood. Alum is classified as a DAMPs inducer (28, 29), but the mode of action of even this widely used adjuvant has not yet been fully described. Because vaccines are administered to healthy people in most cases, the requirement for safety is extremely high, and there is a need to clarify the mechanism of action of adjuvants for their practical use. In this work, we found that HP-β-CD in the respiratory tract mediates immune enhancement via IL-33, in a manner that is distinct from conventional PAMP signaling-based adjuvants. This is the first adjuvant reported to induce humoral immune responses via IL-33.

In our experiment, we performed two types of IL-33 measurement. One is the level of IL-33 in the BALF, which allowed the observation of IL-33 release in the respiratory tract, and the other is the level of IL-33 in lung tissues, which allowed the observation of IL-33 gene expression in the lungs. As shown Figure 1A, the IL-33 level in the BALF of HP-β-CD-treated mice increased and peaked at 6 h after intranasal administration. In contrast, strong IL-33 protein expression was observed in the lung tissues at 24 h after HP-β-CD intranasal administration, and, furthermore, the IL-33 mRNA expression level also increased and peaked at 6 h after HP-β-CD intranasal injection. These results indicate that HP-β-CD can induce both the release of IL-33 protein and the additional gene expression of IL-33 in the lungs. Although alum and CT were also observed to increase the expression of IL-33 at 24 h after intranasal injection, we found that these adjuvants could induce the gene expression of IL-33 in the lungs but were unable to elicit the extracellular release of IL-33. Other studies have concluded that the release of IL-33 in the BALF corresponds with a change to a more active state under inflammatory conditions (30). The other studies have concluded that exogenous IL-33, but not endogenous IL-33, is involved in viral infection (31). These results suggest that the release of IL-33, rather than IL-33 gene expression, might be essential for the adjuvant activity of intranasally administered HP-β-CD.

IL-33 is constitutively expressed in various cells, but the primary source of this cytokine in the lung is airway epithelial cells (16, 32). We observed that the IL-33 expression detected by tissue staining was consistent with pro-SPC-expressing AT2 cells; however, we could not resolve what type of cell death or stress was responsible for the effect of HP-β-CD intranasal administration or how IL-33 is released into the extracellular space. IL-33 is normally evoked by necrotic cell death caused by mechanical or oxidative stress. Conversely, in some situations, such as specific mechanical or oxidative stresses or cell activation through ATP signaling, IL-33 is secreted in the absence of cell death (33–36), whereas upon the commencement of programmed cell death, IL-33 signaling is abrogated (37). Previous studies have reported that DNA release due to cell death indirectly stimulated immune responses through IL-33 (38). It was found that cyclodextrins, particularly β-CDs, extract cholesterol and phospholipids from biological membranes by tapping them into their hydrophobic cores, resulting in the induction of cell death via several pathways (39–42). Thus, further experiments are necessary to determine the mechanisms of IL-33 release after HP-β-CD intranasal administration.

The adjuvant effect of aluminum salts has been extensively studied for many years, and it is thought that upon intratracheal instillation, aluminum salts cause the cell death of alveolar macrophages, which then release IL-1α in the lung, resulting in the induction of type-2 immune responses (21). In our experiments, the induction of IL-1α was also observed after alum intranasal administration (Figure 1A). In contrast, we found that IL-33/ST2 signaling was involved in the adjuvanticity of intranasally administered HP-β-CD. As shown in Figure 1A, HP-β-CD intranasal injection temporarily increased the level of IL-33 but not the level of IL-1α. In the murine lungs, the primary source of IL-33 is alveolar epithelial cells. Thus, we propose that intranasally administered HP-β-CD induces cell damage or death in alveolar epithelial cells, which then release IL-33 into the lung tissues. Moreover, although both alum and HP-β-CD are categorized as DAMPs-inducing adjuvants, it appears that they have distinct mechanisms of adjuvanticity which may be caused from the difference of the target cells to induce DAMPs release. Additionally, we found that the adjuvant effect of subcutaneously administered HP-β-CD, unlike that of intranasally administered HP-β-CD, was not related to IL-33. This difference could be explained by a lower level of IL-33 expression in the skin tissue compared with lung tissues (43, 44). Our results indicate that the IL-33-mediated immune pathway is important for the intranasal administration, but not subcutaneous administration, of HP-β-CD. In support of this, previous studies have demonstrated that mechanisms for protease-dependent sensitization differ between subcutaneous and intranasal administration routes (45). Collectively, these findings indicate that the types of DAMP induced by an adjuvant are distinct between administration routes, resulting in the induction of tissue-specific immune responses.

In this study, we could not identify which type of the cells modulates the immune-enhancing effects by recognizing high levels of IL-33 after intranasal administration of HP-β-CD. Notably, the adjuvant effect induced by HP-β-CD intranasal administration was absent in *St2*^−/−^ mice. ST2 is a receptor of IL-33 that is expressed in various cells, such as Th2 cells, ILC2 cells, mast cells, and regulatory T cells (46). We focused on IL-13, which is produced excessively by ILC2 cells and is critical for T-cell differentiation into Th2 cells. However, in experiments in which HP-β-CD was administered intranasally to *Il13*^−/−^ mice, the local immune response, but not the systemic immune response, was partially attenuated. Furthermore, mast cells may play an essential role in the induction of the antigen-specific mucosal immune responses induced by IL-33 (24, 47). It is the future task for us to identify the involvement of these cells in the immune responses by HP-β-CD inducible IL-33.

We found that the protective ability of an influenza vaccine administered with HP-β-CD against a lethal dose of PR8 influenza virus infection was decreased in *Il33*^−/−^ mice. We also confirmed that the baseline protective immunity is not different between *Il33*^+/−^ and *Il33*^−/−^ mice (data not shown). The antibody titer against HA is also increased by HP-β-CD, and the adjuvant effect of HP-β-CD is abolished in *Il33*^−/−^ mice as well as OVA (Figure S4). These data indicate that IL-33 induced by the intranasal administration of an HP-β-CD-adjuvanted vaccine is also involved in the prophylactic efficacy against influenza virus infection. Previous studies have reported that intratracheal administration of exogenous IL-33 is effective in protecting against influenza infection (31). Although this report concluded that IL-33 is involved in type1 immunity, in our model, IL-33 was involved in antibody production. As it has been reported that intranasal administration of HA antigen and IL-33 in BALB/c mice increased serum IgG1 as well as IgG2a (24), and the influenza infection after intranasal administration of IL-33 in mice produced IL-12p40 in BALF and increased IFN-γ production in the lung (31), the vaccine adjuvant effect of HP-β-CD in flu vaccine may induce type 1 immunity via IL-33. In fact, we have shown previously that IgG2c subtype enhanced by HP-β-CD adjuvanted influenza vaccine in addition to IgG1 (12). Although we have not observed strong type-1 immune responses characterized by the induction of CD4+ Th1 cells and CD8+ T cells by HP-β-CD adjuvanted influenza vaccine, we are keen to investigate the mechanism of the adjuvant effect of HP-β-CD on influenza antigen in near future, as we conducted a human clinical trial, in which HP-β-CD was used as an adjuvant for seasonal 4-valent influenza vaccine (UMIN000028530). Future work should investigate the *in vivo* consequences of an increased antibody titer that is completely dependent on IL-33 as caused by intranasally administered HP-β-CD.

The most intriguing part of this study is the biological significance of the immune system controlled by a single factor of IL-33, such as that induced by HP-β-CD. It is well-known that IL-33 is necessary for parasite protection (48, 49), but this induction of antibody production is completely controlled only by IL-33, while IL-1a, an important inflammatory cytokine in the lung (20, 21), is not involved. In general, IFN and Th1 response play an important role in virus infection, but some viruses circumvent such mechanisms by various means (50). The immune defense mechanism controlled by IL-33 may be a back-up system with an alarm for cell damage by such viruses. A recent studies have reported that IL-33 is involved in the protection of hepatitis viruses and HIV (51, 52). This study seems to be a valuable model for observing antibody production via IL-33 alone.

In conclusion, we found that the intranasal administration of HP-β-CD induces IL-33 release from AT2 cells along with humoral immune responses. These effects were not observed following the subcutaneous administration of HP-β-CD or the intranasal administration of other adjuvants. An improved understanding of the molecular mechanisms of adjuvants will guide research efforts to develop new adjuvants.

## Data Availability Statement

All datasets generated for this study are included in the article/Supplementary Material.

## Ethics Statement

The animal study was reviewed and approved by National Institutes of Biomedical Innovation, Health and Nutrition.

## Author Contributions

SK, TK, EK, SI, and KI designed the study. SK, TK, MM, TS, TH, HM, KM, HS, and EK performed the experiments. SK, TK, SI, KO, EK, and KI interpreted the data and drafted the manuscript. All authors approved the final version.

### Conflict of Interest

The authors declare that the research was conducted in the absence of any commercial or financial relationships that could be construed as a potential conflict of interest.
