# Supplementary material for: IL-33 Is Essential for Adjuvant Effect of Hydroxypropyl-β-Cyclodexrin on the Protective Intranasal Influenza Vaccination
Source: Front Immunol. 2020 Mar 6;11:360. doi: 10.3389/fimmu.2020.00360 (PMC7069475; doi:10.3389/fimmu.2020.00360)
Supplement: Supplementary file 1 [file Data_Sheet_1.docx]

Supplementary Material

*Shingo Kobari^1^**^,2†^, Takato Kusakabe^1,3,4†^, Masatoshi Momota^1,3^, Takayuki Shibahara^1,3^, Tomoya Hayashi^1,5,8^, Koji Ozasa^1,2^ , Hideaki Morita^6^, Kenji Matsumoto, Hirohisa Saito^6^, Shuichi Ito^2^, Etsushi Kuroda^1,7^, Ken J. Ishii^1,3,4,8,‡*^*

*^1^ Laboratory o Adjuvant Innovation, Center for Vaccine and Adjuvant Research, National Institutes of Biomedical Innovation, Health and Nutrition (NIBIOHN), Osaka, Japan*

*^2^ Department of Pediatrics, Yokohama City University Graduate School of Medicine, Kanagawa, Japan*

*^3^ Laboratory of Mock-up Vaccine Project, Center For Vaccine and Adjuvant Research, National Institutes of Biomedical Innovation, Health and Nutrition (NIBIOHN), Osaka, Japan*

*^4^ Laboratory of Vaccine Science, WPI Immunology Frontier Research Center (IFReC), Osaka University, Osaka, Japan*

*^5^ Division of Vaccine Science, Department of Microbiology and Immunology, The Institute of Medical Science, The University of Tokyo, Tokyo, Japan*

*^6^ Department of Allergy and Clinical Immunology, National Research Institute for Child Health and Development, Tokyo, Japan.*

*^7^ Department of Immunology, Hyogo College of Medicine, Hyogo, Japan*

*^8^ International Research and Development Center for Mucosal Vaccines, The Institute of Medical Science, The University of Tokyo, Tokyo, Japan*

***†*** *These authors have contributed equally to this work*

** Correspondence:
Ken J Ishii, M.D.,Ph.D.*[*kenishii@ims.u-tokyo.ac.jp*](mailto:kenishii@ims.u-tokyo.ac.jp)*,* [*kenishii@biken.osaka-u.ac.jp*](mailto:kenishii@biken.osaka-u.ac.jp)

# *Supplementary Figures and Tables*

## *Supplementary Figures*

***
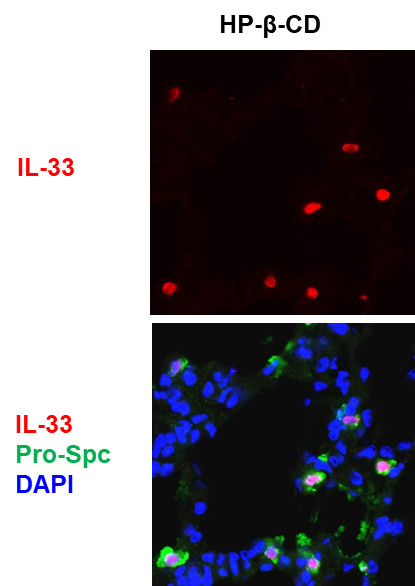
***

***Figure S1. Nucleic acid signaling was not involved in IL-33 expression after intranasal administration of HP-β-CD.*** *Tbk1^−/−^/Tnf^−/−^ mice were nasally administered a single dose of 10% HP-β-CD. Immunohistochemistry of IL-33 (red) and Pro-SPC (green) in the lungs at 24 h after intranasal administration of HP-β-CD; the sections were co-stained with DAPI (blue).*

*
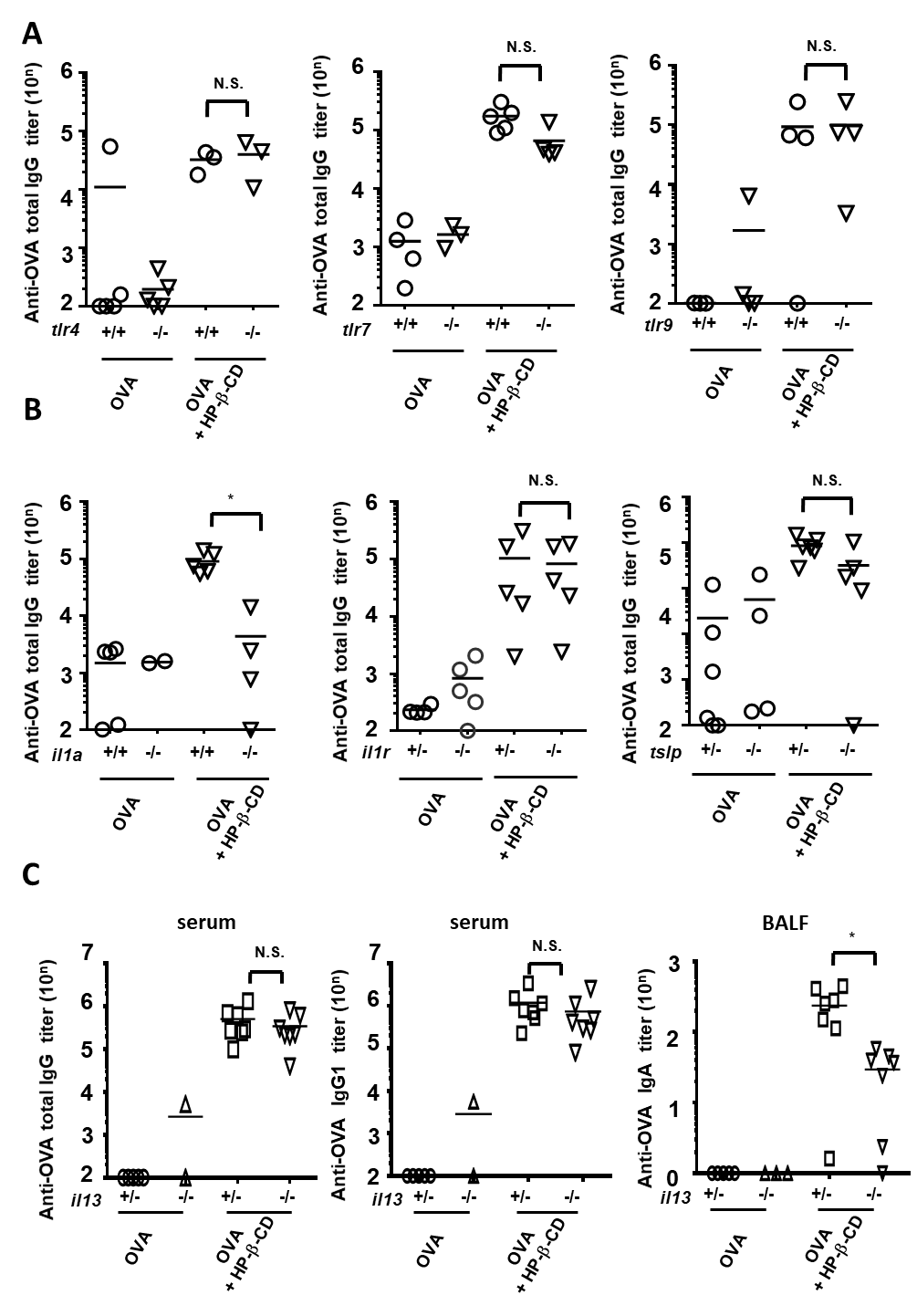
*

***Figure S2.*** ***Antibody responses in BALF following intranasal HP-β-CD administration in various knockout mice.*** *Tlr4^−/−^, Tlr7^−/−^, Tlr9^−/−^, Il1a^−/−^, Il1R^−/−^, Tslp^−/−^, Il13^+/−^, or Il13^−/−^ mice were immunized twice with OVA ± 10% HP-β-CD via intranasal administration on days 0 or 7. Anti-OVA total IgG, IgG1, and IgG2c antibody responses in the serum and IgG1 and IgA in the BALF were measured seven days after the last immunization. *p < 0.05 (Mann-Whitney U test).*

*
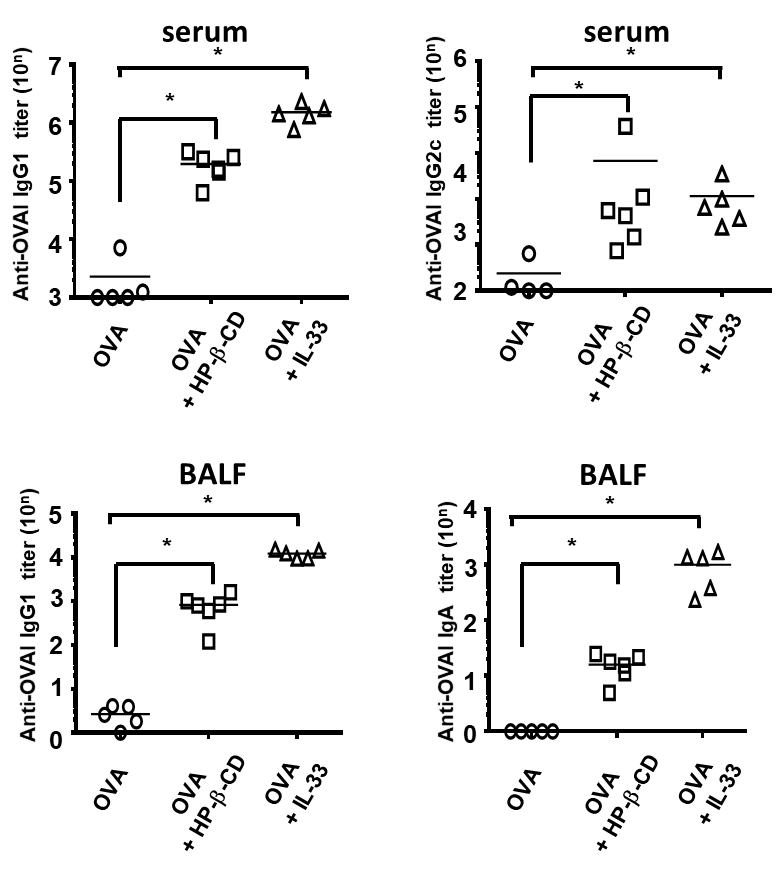
*

***Figure S3.*** ***Intranasal administration of recombinant IL-33 shows a mucosal adjuvant as well as HP-β-CD.*** *Mice were immunized twice with OVA ± 10% HP-β-CD or 150 ng recombinant IL-33 via intranasal administration on days 0 or 7. Anti-OVA-IgG1 and IgG2c antibody responses in the serum and IgG1 and IgA in the BALF were measured seven days after the last immunization. *p < 0.05 (Mann-Whitney U test).*

*
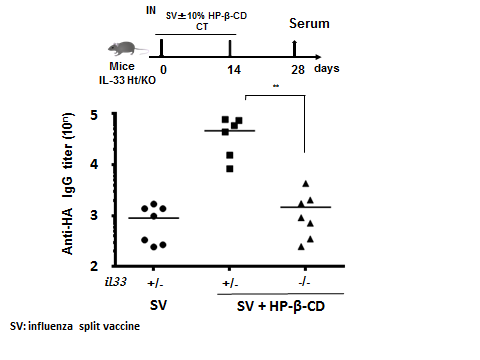
*

***Figure S4. HP-β-CD was also able to increase antibody titers against influenza vaccine.*** *Il33^+/-^ or Il33^-/-^ mice were immunized twice (days 0 and 14) with SV (1ug) or SV (1ug) + HP-β-CD by intranasal administration (n = 6-7). Two weeks after the last immunization, anti-HA specific total IgG titers were measured. **p < 0.01(Mann-Whitney U test)*
